# The Role of Meta-cognition in Students' Addiction Potential Tendency

**DOI:** 10.5812/ijhrba.9355

**Published:** 2014-03-15

**Authors:** Nader Hajloo, Hasan Sadeghi, Karim Babayi Nadinloei, Zahra Habibi

**Affiliations:** 1 Department of Psychology, University of Mohaghegh Ardabili, Ardabil, IR Iran; 2 Young Researchers Club and Elites, Islamic Azad University Branch Science and Research of Ardabil, Ardabil, IR Iran

**Keywords:** Behavior, Addictive, Students, Cognition

## Abstract

**Background:**

Addiction for narcotics is a dangerous reality, especially in teenagers and young persons, and is one of the most important socioeconomic and health problems, threatens the human society and leads to social stagnancy in various aspects.

**Objectives:**

The present study aimed at determining Meta-cognitive relationship with addiction potential tendency in students and determining the distribution of each Meta-cognitive dimension in predicting addiction potential tendency.

**Materials and Methods:**

The descriptive-correlative method was used for conducting this research. The research target population included all students of Ardebil Mohaghegh University. A sample of 380 subjects was selected randomly from this demographic population by cluster multistage sampling. We used Weed and colleagues’ questionnaire of addiction to assess addiction potential tendency, and the Meta-cognitive scale of Wells and colleagues for evaluating Meta-cognition. Data gathered were analyzed by multifold Regression via simultaneous entrance method.

**Results:**

The rate of students’ addiction potential tendency can be predicted by having characteristics of their Meta-cognition. It also appeared that among the five parameters of Meta-cognition, the role of three parameters required for controlling thoughts, uncontrollability and risk, and positive beliefs about anxiety were of great importance in predicting addiction potential tendency in students.

**Conclusions:**

The results of this research have important implications regarding the attention of students’ counselors to Meta-cognitive dimensions in order to prevent students’ tendency for addiction.

## 1. Background

Addiction for narcotics is a dangerous reality, especially in teenagers and young persons, and is one of the most important socioeconomic and health problems that results in real threat for human society and leads to social stagnancy in different aspects (1). Surveys about drug abuse among students resulted different data but most findings focus on increase of illegal drug abuse among students (2). Seventy three percent of students among drug abusers were non-native, whom 45% of them lived in a dormitory (3).

Since youth and adolescence is a time for experiencing and personal selections and personal identity is shaped in this period, youths and adolescents are more vulnerable to drug abuse and dangerous behaviors (4). For this reason, recognizing effective factors in preventing and rescuing youths from dangerous drugs’ abuse is of great importance. In researches related to drug dependency, besides explaining different dimensions of this problem, Researchers have tried to discover adiction predictor variables. One of these important variables is Meta-cognitive factor (5).

Meta-cognitive is a multifaceted concept and includes knowledge, beliefs, processes and guidelines which evaluates and controls cognition (6). Meta-cognition is knowledge or cognitive process involved in evaluation, reviewing or controlling cognition (7). Most theorists distinguish the two Meta-cognitive aspects, that is, Meta-cognitive knowledge and Meta-cognitive surveillance. Meta-cognitive knowledge is individual’s information about self-awareness and learning guidelines (that these guidelines have influence on them); and, Meta-cognitive surveillance is scope of administrative functions like attention, controlling, investigating, planning and detecting errors in performance (8).

Researchers have found direct or indirect relations between Meta-cognition and psychological disorders such as obsession (9), hypochondria (10), exam anxiety (11) negligence (7, 11) alcoholic drinking problems (11) and drug dependency (12). Two main reasons about inevitable relation between Meta-cognition and alcoholic drinking and addiction are:

1. Using alcohol and drugs have been conceptualized as a guideline for regulating emotions and negative recognitions.
2. Excitement features (anxiety and depression) linked with using alcohol and drugs and according to administrative function theory of self-regulation, excitement confusions linked with inconsistency of Meta-cognitions.

Therefore, it is possible that the Meta-cognitive factors have direct relation with excitements and drug abuse, and indirect correlation with alcoholic drinking by excitement disorders. Wells et al. conducted the first studies regarding the role of Meta-cognitive beliefs in drug dependency showing relation between these two factors (12). Specifically, Spada et al. (11) showed that there is positive and significant relation between three of Meta-cognitive dimensions, i.e. positive thoughts about anxiety, negative thoughts about anxiety, and beliefs about cognitive confidence and drug dependency; but two other Meta-cognitive dimensions, i.e. beliefs about controlling thoughts and cognitive self-awareness had marginally linked to drug dependency.

Also Saeed et al. (5) showed that among Meta-cognitive operatives, the negative thoughts’ element about uncontrollability and risk in predicting disorder of drug dependency had higher contribution. Wells (12) and Haji-alizadeh et al. (13) also reported that four of cognitive dimensions (positive beliefs about anxiety, negative beliefs about anxiety and beliefs about cognitive confidence and believing the need for controlling thoughts) have significant positive relation with alcohol drinking.

As mentioned, different surveys examined the role of Meta-cognitive beliefs in dependency on drugs and showed that Meta-cognition is related to drug dependency. Furthermore, due to importance of students’ role in future of any country, predicting readiness for addiction and performing required actions for prevention are necessary. Therefore, the aim of this research is to explain Meta-cognitive relation with readiness for addiction in students and to determine contribution of each Meta-cognitive dimensions in predicting readiness for addiction.

Given that, the issue of addiction is a serious threat to individuals, families and society, and also regarding lack of enough researches on role of Meta-cognition in readiness to addiction, the present study investigated the role of Meta-cognition in students’ readiness for addiction. Another important study of Meta-cognition in addiction is due to its influence in the thoughts, behavior and attitudes of individuals. The importance of preventive and therapeutic aspects of Meta-cognitive beliefs in addiction is what is essential in this study. It aims to reveal the role of predictively of Metacognitive beliefs in tendency to addiction. Research on the factors associated with drug dependence can be effective in identifying psychological variables in addiction.

## 2. Objectives

The present study aimed at determining Meta-cognitive relationship with addiction potential tendency in students and determining the distribution of each Meta-cognitive dimension in predicting addiction potential tendency.

## 3. Materials and Methods

This descriptive-correlative method was done on. All bachelor students of Mohaghegh Adabili University who were studying in the 2011-2012 school-year (12000 students). Based on Morgan table, a sample including 380 subjects was selected by multi-stages clustered randomly from 5 faculty of Mohaghegh Adabili University. Therefore, two departments were selected from each faculty, and two educational courses from each department (10 courses in total) were randomly selected. The participants of study were undergraduates in agriculture, animal science, chemistry, biology, Persian literature, geography, civil engineering, mechanics, mathematics and statistics courses. The subjects were aged between 18-45 and mean age of 27.3 ± 3.12 years. Single subjects consisted of 64.1% and 35.9% were married. The obtained data were analyzed by simultaneous entrance method via applying Multiple Regression. 

### 3.1. Instruments

#### 3.1.1. Meta Cognition Questionnaire

The short forms of Metacognitive questionnaires MCQ-30 (14) were applied for evaluating students’ Meta-cognitive beliefs. This questionnaire is a self-report scale of Meta-cognitions and contains 30 questions and 5 subscales which is graded as four-choices with Likert scale from "I don’t agree" = 1 to "I completely agree" = 4. This questionnaire composed of five separate variables: positive beliefs about worry, negative beliefs about riskiness and uncontrollability, lack of cognitive self-confidence, beliefs about the need for controlling thoughts, and cognitive self-awareness. The Cronbach’s Alpha coefficient of this questionnaire and its parameters are reported from 0.72 to 0.93. The correlation coefficient of this questionnaire are somewhat related to was some level with questionnaire of facial-state anxiety of Spielberg (r = 0.53), Pen’s state depression questionnaire (r = 0.54) and Bodow’s obsessive compulsive questionnaire (r = 0.49). In present study, the alpha coefficient of Metacognitive questionnaire was 0.88.

#### 3.1.2. The Questionnaire of Addiction Potential Tendency

For measuring addiction tendency, the addiction preparation subscale (APS), questionnaire of addiction measurement of Weed et al. (15), composing of three addiction preparation subscale (APS): the addiction acceptance scale and scale of alcohol propensity or the wine Bargy of Mac Andrew were used. Addiction measurement questionnaire in Iran has been normalized (16). The real version of addiction preparation subscale (APS) contains 39 questions. Answers given for each scale material are “yes” or “no”. The APS normalized versions for Iranian high school students (1) has 36 items, based on Mohammadi et al. (2) study. The scores from 1-18 shows very low tendency for addiction; scores 19-20 shows low tendency, scores 21-22 shows average tendency, scores 23-24 shows high tendency, and score 25 and higher shows very high tendency to. In present study, the Alpha coefficient of questionnaire for addiction potential tendency was 0.91.

## 4. Results

With regard to the elongation and choleric of Table 1, single variable sample scores being studied are normally distributed

**Table 1.** Descriptive Indicators of Addiction Potential Tendency and Meta-cognition Components

	Min	Max	M	Sd	Kurtosis	Skewness
**Readiness for addiction**	9	52	19.89	6.48	0.71	0.65
**Need to control thoughts**	7	23	15.36	3.16	0.33	0.04
**Cognitive self-awareness**	6	20	12.51	2.85	0.23	0.26
**Uncontrollability and danger**	8	44	16.63	4.29	0.07	0.019
**Positive thoughts about anxiety**	8	22	14.6	3.38	0.3	0.48
**Cognitive confidence**	7	34	16.44	3.77	0.25	0.04

Before the regression analysis of the addiction potential tendency on components of Meta-cognition, correlation coefficient of students' readiness for addiction with components of Meta-cognition was calculated (Table 2).

**Table 2.** Correlation Coefficient of Students Addiction Potential Tendency With Components of Meta-cognition (N = 380)^a^

	NC	CSA	UnD	PTA	CC
**Readiness for addiction**	-0.41 ^b^	-0.17 ^c^	-0.39 ^b^	-0.52 ^b^	-0.15 ^c^

^a^ Abbreviations: NC, need to control thoughts; CSA, cognitive self-awareness; UnD, uncontrollability and danger; PTA, positive thoughts about anxiety; CC, cognitive confidence

^b^ P < 0.05

^c^ P < 0.01

**Table 3.** Multiple Regression Analyzing of Addiction Potential Tendency Based on Metacognitive Components

	B	SE	Beta	t	R^2^	R	F
					0.31	0.55	2.54 ^a^
**Need to control**	0.24	0.28	0.20	2.09 ^a^			
**Cognitive awareness**	0.07	0.31	0.06	1.06			
**Uncontrollability and danger**	0.21	0.19	0.19	1.97 ^a^			
**Positive thoughts about depression**	0.38	0.25	0.35	3.53 ^a^			
**Cognitive confidence**	0.04	0.20	0.04	0.62			

^a^ P < 0.01

The separate standard multi-regressions (simultaneous entrance method) were used to determine the rate of determined variance of addiction potential tendency (Table 3). Before conducting regression analysis, an initial analysis took place to examining assumptions of multivarious Regressions. The results represented the normality of multivarious scores (residuals were distributed normally around predicted scores of addiction potential tendency. The relation among variables was linear (residuals had a direct linear relation with predicted scores of readiness for addiction). There was no multivarious co-linear correlation between independent variables, i.e Meta-cognitive parameters was lower than 0.7. There frequencies were similar regarding (that the residuals’ variance around predicted scores for all predicted scores was the same.

The result of multivarious regression analysis of addiction potential tendency toward predicted variables (Table 2) showed that 31 percent of all variances of addiction potential tendency by inserted variables in model were determined. ANOVA analysis on this model showed the significance of complete model: (F (5 and 77) = 2.54 and P < 0.01). To be aware of each predicting variable’s distribution in determining variable variance of addiction potential tendency, the Beta coefficient was examined.

The results showed that the single distribution of three variables needed for thought controlling, uncontrollability and danger, positive beliefs about anxiety are statistically significant (P < 0.01). Meanwhile, positive beliefs about anxiety and need to control thoughts assigned the highest distribution in determining variance (P < 0.01).

## 5. Discussion

The aim of the present research was to determine the relation of Meta-cognition with addiction potential tendency in students and to determine the share of each Meta-cognitive dimension in predicting the readiness for addiction. Findings showed that the more the person has inefficient Meta-cognitive beliefs, the higher the possibility of inclination toward dependency to narcotics or becoming caught in this disorder. These findings are comparable to with those of Wells (12), Spada et al. (11), Toneatto (17), Haji-alizadeh et al. (13), Saeed et al. (5).

Also among the Meta-cognitive factors, three factors were required for controlling thoughts, uncontrollability and risk and positive beliefs about anxiety in predicting readiness for addiction in students rather than two other factors; cognitive awareness and confidence awareness are considered significant.

Meta-cognitive beliefs refer to beliefs which individual holds about one’s thoughts and its processes. The maxim of S-Ref’s theory is such that these beliefs and experiences influence the continuousness and abnormality of countering behaviors. Based on S-Ref’s theories (Self-adjusted administrative performances), psychological disorders continues via complying with abnormal strategies, like repetitive thoughts (anxiety and mental rumination), threatening supervision behavior, avoidance and preventing thoughts which fails in moderating malfunctioning of own beliefs and increases the possibility of reaching negative information about oneself (8). These factors form the syndromes of cognitive attention (8). The cognitive attention syndrome derives from individual Meta-cognitive awareness that is activated in difficult situations and by activation of cognitive attention syndrome, which itself derives from cognitive beliefs. The strategies to be complied with (such as drug abuse) are activated and hence the ground of creation and continuous of drug dependency disorder is risen in individuals.

Furthermore, based on underwent investigations, Meta-cognitive theories contributing understanding addiction potential tendency, might be considered as a behavioral guideline in controlling unexpected behaviors and excitements; informed by negative beliefs about cognition (beliefs about controlling unexpected thoughts and excitements) (18). Individuals experiencing negative excitement as anxiety and depression may expect to use alcohol, knowing that alcohol leads to reduction of worry and aggression resulted from anxiety; Spada et al. reported (18).

The study of Toneatto (17) approved the positive Metacognitive beliefs in addiction potential tendency and reminded that beliefs reflect the positive Meta-cognition to drug use tendency, the suitability and usefulness of psychiatric drugs in adapting excitements and cognitive states, and negative Meta-cognitive beliefs lead to destructive effects and uncontrollability in use of drugs.

Two of Meta-cognitive dimensions (positive ideas about anxiety and beliefs about cognitive confidence) show the importance on Meta-cognition in memory inefficiency awareness. Overall, these variables can reflect the degradation of trust in agreeing, the need to predict problems (complete worry) and cognitive control; and if this hypothesis becomes approved, these beliefs will be guidance for low cognitive confidence. Therefore, it can partake in dependency for cigar and drugs, due to increase of inner cognitive confidence; which leads to improvement, agility, and fastness of information processing and verbal memory. In addition, drug use must reduce Meta-cognitive irritation. Besides, it is possible that beliefs about uncontrollability of thoughts can reflect tendency for unread thoughts related to enthusiasm or temptation and these are signs for beliefs related to tempted thoughts which must be controlled; otherwise, they will control the behavior. Thus, if strategies that are used for controlling thoughts are abnormal (mental rumination, thought rejection ad finally narcotics), it will lead to increasing negative thoughts about oneself, which finally leads to anxiety and depression; and based on underwent investigations, anxious and depressed people allude to drugs in order to lessen their anxiety and worries.

According to findings of this study and previous researches, further importance to Meta-cognitive factors in etiology and treatment of substance abuse disorders should be given. Due to relationship between Meta-cognitive factors, the need to control thoughts, uncontrollability, and positive beliefs about worry have significant contribution in prediction of addiction potential tendency. It is necessary to pay more attention to these factors. Based on the Meta-cognitive model, treatment process of substance abuse disorder should not undermine the thoughts and beliefs of addicted peoples or put them into reality testing, because such efforts are a waste of time. Rather, the goal of treatment should be to evaluate and change in how addicted people respond to drug in their beliefs in the cognitive processes.

The research faced with some limitations: conducting the research among students makes it difficult to generalize the results other strata; hence it is recommended to test this relationship among other social classes in further studies. Furthermore, this research was a descriptive-correlative study, therefore determining cause and effect relations between research variables is improvidence whatsoever. The present research was depended on reporting individual's Meta-cognitive and addiction potential tendency, which may not be a reflection of people's real lives. In the next studies, this limitation can be overcome by applying reports of friends and peers or by designing experimental patterns.

Irrespective of the above-mentioned methodological limitations, the results of this research besides supporting other similar studies and elaborating their findings, renders better research evidences for researchers. Since the role of psychological variables in readiness for addiction is so complex, exact judgment about Meta-cognitive role in addiction potential tendency cannot be done merely by relying on the results of this research; thus, conducting various researches in this field is necessary. It is recommended that in future researches the role of socio-economic status and other psychiatric disorders in predicting addiction potential tendency should be evaluated.

## References

[1] Khangani Z, Fakhraiy N, Badri R (2011). The role of meta cognition variables, meta cognition dimention and emotions in using of materials.. Res Behave Sci..

[2] Mohammadi A, Aghajani M, Zehtabvar G (2011). The relationship between addiction, resilience and emotional variables.. Psychiat Clin Psychol..

[3] Bolhari J, Taremian F, Piravi H (2007). Prevalence of Substance use among students in Tehran and evaluation of risk and protective factors.

[4] Rotheram-Borus M, Miller S, Koopman C, Haignere C, Selfringe C (2002). Adolescents living safely, AIDS awareness, attitudes and actions..

[5] Saeed O, Pour-ehsan S, Aslani J, Zargar M (2011). The role of thought prevention, meta cognition factors and negative emotion in prodecting disorder of dependance to meterials.. Sci Addict..

[6] Moses L, Baird JA (2002). Analysis on Meta cognition variables..

[7] Sadeghi H (2011). The Study of Relationship Between Meta Cognition Beliefs and Procrastination Among Students of Tabriz and Mohaghegh Ardabili Universities.. Procedia Soc Behav Sci..

[8] Wells A (2002). Emotional disorders and metacognition: Innovative cognitive therapy..

[9] Emmelkamp PM, Aardema A (1999). Metacognition, specific obsessive–compulsive beliefs and obsessive–compulsive behaviour.. Cli Psychol Psychother..

[10] Bouman TK, Meijer KJ (1999). A preliminary study of worry and metacognitions in hypochondriasis.. Clin Psychol Psychother..

[11] Spada MM, Hiou K, Nikcevic AV (2006). Metacognitions, emotions, and procrastination.. J Cogn Psychother..

[12] Wells A (2011). Metacognitive therapy for anxiety and depression..

[13] Haji-alizadeh K, Bahrinain S, Naziri G, Modares-Gharvani M (2009). The Role of Cognitive Variables, Metacognitive Dimensions and Emotions in Substance Abuse Behaviors.. Adv Cogn Sci..

[14] Wells A, Cartwright-Hatton S (2004). A short form of the metacognitions questionnaire: properties of the MCQ-30.. Behav Res Ther..

[15] Weed NC, Butcher JN, McKenna T, Ben-Porath YS (1992). New measures for assessing alcohol and drug abuse with the MMPI-2: The APS and AAS.. J Pers Assess..

[16] Minooei M, Salehi E (2009). Valuation of reliability, validity and normative issue APS test toidentify individuals predisposed to addiction.. J Res Add..

[17] Toneatto T (1999). Metacognition and substance use.. Addict Behav..

[18] Spada MM, Nikcevic AV, Moneta GB, Wells A (2007). Metacognition as a mediator of the relationship between emotion and smoking dependence.. Addict Behav..

